# Expression of E-Cadherin in Epithelial Cancer Cells Increases Cell Motility and Directionality through the Localization of ZO-1 during Collective Cell Migration

**DOI:** 10.3390/bioengineering8050065

**Published:** 2021-05-11

**Authors:** Song-Yi Park, Hwanseok Jang, Seon-Young Kim, Dasarang Kim, Yongdoo Park, Sun-Ho Kee

**Affiliations:** 1 Department of Microbiology, College of Medicine, Korea University, Seoul 02841, Korea; songyip3507@korea.ac.kr (S.-Y.P.); ksy0817@korea.ac.kr (S.-Y.K.); darang233@korea.ac.kr (D.K.); 2 Department of Biomedical Sciences, College of Medicine, Korea University, Seoul 02841, Korea; kevin14@korea.ac.kr or

**Keywords:** E-cadherin, ZO-1, cell patterning, motility, directionality, cancer metastasis

## Abstract

Collective cell migration of epithelial tumor cells is one of the important factors for elucidating cancer metastasis and developing novel drugs for cancer treatment. Especially, new roles of E-cadherin in cancer migration and metastasis, beyond the epithelial–mesenchymal transition, have recently been unveiled. Here, we quantitatively examined cell motility using micropatterned free edge migration model with E-cadherin re-expressing EC96 cells derived from adenocarcinoma gastric (AGS) cell line. EC96 cells showed increased migration features such as the expansion of cell islands and straightforward movement compared to AGS cells. The function of tight junction proteins known to E-cadherin expression were evaluated for cell migration by knockdown using sh-RNA. Cell migration and straight movement of EC96 cells were reduced by knockdown of ZO-1 and claudin-7, to a lesser degree. Analysis of the migratory activity of boundary cells and inner cells shows that EC96 cell migration was primarily conducted by boundary cells, similar to leader cells in collective migration. Immunofluorescence analysis showed that tight junctions (TJs) of EC96 cells might play important roles in intracellular communication among boundary cells. ZO-1 is localized to the base of protruding lamellipodia and cell contact sites at the rear of cells, indicating that ZO-1 might be important for the interaction between traction and tensile forces. Overall, dynamic regulation of E-cadherin expression and localization by interaction with ZO-1 protein is one of the targets for elucidating the mechanism of collective migration of cancer metastasis.

## 1. Introduction

Cell migration is essential for animal growth and physiological activity; the movements of various cell types have been investigated [1] and many cells in tissues such as developing neural and vasculatures or epithelial cells in the wound healing are known to migrate collectively rather than individually [2]. In addition, the spread of tumor cells in epithelial tissues such as squamous carcinoma is known to be associated with the collective migration of cells [2,3,4]. The ability of cancer cells to migrate is significantly associated with the degree of cancer malignancy [3]. The dynamics of collective migration are based on the interplay of cell–cell junctions within the cell population. Accordingly, collective migration of cancer cells reflects movements of epithelial cells and can occur in the absence of the epithelial–mesenchymal transition (EMT), whereas individual cell migration occurs with the EMT like the movement of mesenchymal cells [5,6,7].

Cell-cell adhesion in cancer cells plays an important role in cancer biology. Interest in adhesion molecules and their ability to predict cancer progression has increased as a result of their role in the invasion and metastasis of cancer cells [8]. Currently, more than 100 cell adhesion molecules have been identified and these molecules are interrelated in function despite their differences in biochemical and genetic characteristics [9]. Loss of cell-cell adhesion is caused by changes in the expression of adhesion proteins, which plays an important role in infiltration and metastasis [10,11]. A major component of adherent junction (AJ), E-cadherin is well known as a negative regulator of Wnt signaling, is also categorized as a tumor suppressor because of its ability to inhibit cell migration and the carcinoma-related EMT in primary tumors [12]. However, several reports have suggested that E-cadherin re-expression may promote cell survival within metastatic sites [13,14,15].

Tight junctions (TJs) are one type of intercellular junction observed in the human epidermis; these junctions connect adjacent cells located at the cell gap of the epidermal granular cell layer, functioning as a barrier to control the movement of electrolytes and water [16]. ZO-1, a protein component of TJs, is highly correlated with the regulation of epidermal adherent junction (AJ)s [16,17,18]. The N- and C-terminal domains of ZO-1 interact directly with α-catenin and actin filaments, respectively; these interactions serve as a bridge between the cadherin/catenin complex and the actin cytoskeleton [19]. ZO-1 also interacts with anchor membrane proteins such as claudins, occludin, and junctional adhesion molecules (JAMs), linking them to the actin cytoskeleton [20]. Recent studies have linked TJs with the transcription of cancer-related genes, inhibition of tumor metastasis, and regulation of cell proliferation [17,21,22].

Monitoring cell migration and analysis of its migratory activity in vitro is a fascinating tool to understand the behavior of collective cell migration. Microfabrication technologies such as patterning extracellular matrix (ECM) proteins, stencils for confining cell clusters, and microgroove or nano-topology enabled us to understand the cellular behavior during collective cell migration. Especially, the free edge migration model using a micro-sized stencil is a simple and ideal tool to investigate the collective cell migration [23,24]. Analysis of cellular kinematics and force distribution based on patterning technology showed that cell-cell junction such as cadherin and redistribution of vinculin is important to control the collective cell migration [24,25].

In this study, we investigated the collective cell migration of epithelial cancer cells using micropatterned free edge migration models. Analysis of speed and directionality of migration provides a multi-lateral analysis of the migratory activity of cells. Based on this model, we evaluated the function of cell-cell junctions by E-cadherin during collective cell migration and studied the basic mechanism of TJ formation by ZO-1 and CLDN7 in the directionality of boundary cells within the cell collectives.

## 2. Materials and Methods

### 2.1. Cell Culture

AGS cells isolated from gastric cancer tissue were purchased from Korea Cell Line Bank in 2003. The frozen stock was thawed and the growth characteristics, shape, and mycoplasma contamination were checked after freezing prior to the experiment. EC96 cells were derived from AGS cells after transfection of E-cadherin cDNA. E-cadherin-expressed AGS cells were selected with neomycin and several rounds of single-cell cloning. The establishment of EC96 cells was described previously [26]. AGS and EC96 cells cultured in Dulbecco Modified Eagle Medium (DMEM) (Lonza, Walkersville, MD, USA) with high glucose and L-glutamine, supplemented with 10% fetal bovine serum (FBS) (Corning, Manassas, VA, USA), 10,000 U/mL penicillin, and 10,000 μg/mL streptomycin (Lonza, Walkersville, MD, USA), and maintained in humidified incubators at 37 °C with 5% CO_2_.

### 2.2. Transient Transfection with siRNAs and Stable Knockdown Cell Line Generation

Lentivirus expressing shRNA for pLKO-shZO-1 or pLKO-shCLDN7 were purchased from sigma. To produce lentivirus expressing shRNA co-transfected with the lentivirus packaging plasmids (psPAX2, pMD2G, and VSV-G) into 293T cells. The virus-containing cell culture supernatant was harvested, filtered through a 0.22-μm pore-size filter, and used to infect EC96 cells. To generate stable ZO-1 and CLDN7 knockdown cells, infected cells were selected by puromycin (2 μg/mL), and several rounds of single-cell cloning. The establishment of E-cadherin knockdown EC96 stable cell lines were described previously [27].

Specific siRNAs, targeting ZO-1, CLDN1 CLDN7, and non-specific control siRNAs were purchased from Santa Cruz Biotechnologies. For transient transfection, cells were seeded at a density of 5 × 10^4^ cells/mL in an antibiotic-free medium, and siRNAs were transfected using the transfection reagent (Santa Cruz Biotechnology, Dallas, TX, USA), according to the manufacturer’s instructions. After incubation for 48 h, the cells were analyzed using in vitro wound-healing assay.

### 2.3. Fabrication of Polydimethylsiloxane (PDMS) Stencils

PDMS stencils for circular cell islands patterning were produced by soft lithography using PDMS (Sylgard 184, Dow Corning, Midland, MI, USA) following the methods reported in previous studies [24]. In brief, a PDMS pre-cured solution mixing 10:1 ratio of prepolymer to curing agent was poured over the SU-8 master mold (outsourcing, Amed, Seoul, Korea) with a thickness of 200 μm and cured in a dry oven at 85 °C for 2 h. After curing, a thin PDMS film with hole arrays (diameter = 1 mm) was trimmed with a 14 mm diameter punch. The resulting PDMS stencils were sterilized with autoclaving.

### 2.4. Micropatterning of Cell Islands

**Figure 1:**
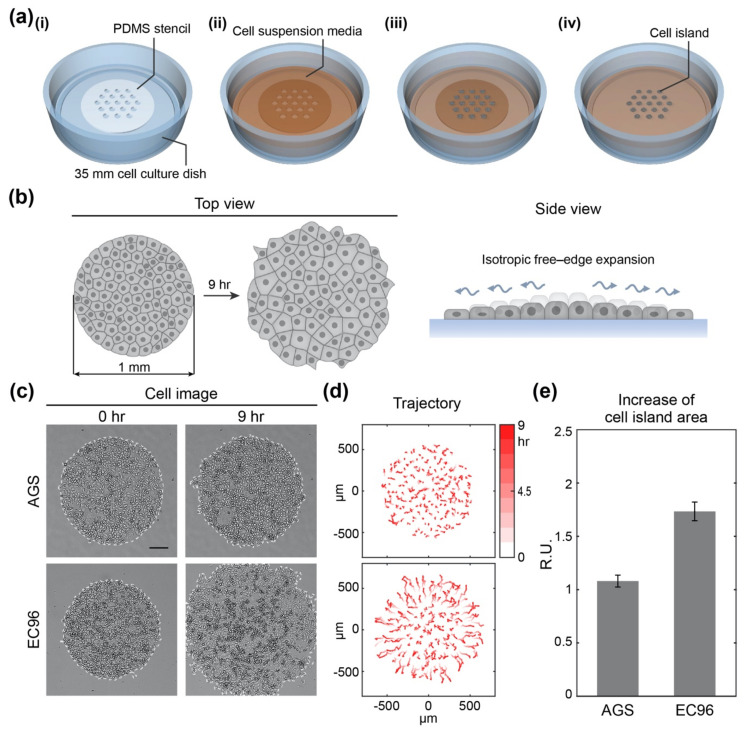
Experimental set-up of the isotropic free-edge expansion model and expansion of AGS and EC96 cell islands. (**a**) Schematic of circular-shaped cell island patterning for the isotropic free-edge expansion model; (i) placing a PDMS stencil in a 35 mm cell culture dish; (ii) adding cell suspension media over the stencil in the dish; (iii) incubating the dish for the cells to settle down within the holes of the stencil; (iv) removing the stencil to release the cell island to begin the expansion assay. (**b**) Schematic of the experimental model for isotropic free-edge expansion. (**c**) Bright-field images of AGS and EC96 cell islands at 0 h and 9 h after release (scale bar = 200 μm). (**d**) The trajectory of the cells within the AGS and EC96 cell islands. The stronger red color of the arrows indicates more time passed. (**e**) Relative increase rate of the AGS and EC96 cell islands. The error bars represent the standard errors calculated from separate assays on each group (*n* = 5).

The prepared PDMS stencils were immersed in a 2% (*w*/*v*) Pluronic F-127 solution (Sigma-Aldrich, St. Louis, MO, USA) diluted with phosphate-buffered saline (PBS) (Thermo Fisher Scientific, Waltham, MA USA) and stored in a 37 °C incubator for 1 h. After washing the stencil three times with PBS, the stencil dried in ambient air was placed on the bottom of a 35 mm cell culture dish (SPL life sciences, Pocheon, Korea) (Figure 1a). We filled 4 mL of PBS into the dish, removed air bubbles in the stencil hole by gentle pipetting, and removed the PBS surrounding the stencil by suction. We added 2 mL of cell suspension media at a concentration of 1 × 10^5^ cells/mL in DMEM over the stencil and stored the samples in an incubator overnight. When the cells settled on the bottom of the dish and formed cell islands along with the hole shape of the stencil, we removed the stencil and rinsed it with DMEM gently to remove cell debris.

### 2.5. Live Cell Image Acquisition: Time-Lapse Microscopy

Bright-field images for cells were acquired every 10 min for up to 9 h using a JuLI stage live cell imaging system (NanoEnTek, Seoul, Korea) with a 4× magnification objective lens (Olympus, Tokyo, Japan) housed within an incubator.

### 2.6. Cell Velocities and Trajectories

The acquired bright-field cell images were numerically transformed and quantitatively analyzed using custom codes written in MATLAB (MathWorks Inc., Portola Valley, CA, USA). The codes used in this study to extract cell displacements and velocities are based on the particle image velocimetry (PIV) code used in the previous study [24,25]. The consecutive cell island images of 1216 × 1216 pixels (1070 × 1070 μm) taken at 10 min intervals were cross-correlated using unit pixel subset windows (interrogation windows, IWs) of 64 × 64 pixels at a spacing of 16 pixels (14 μm). The displacement vectors of the grid of 73 × 73 interrogation window (IW)s obtained at each time interval were converted into instantaneous cellular velocities by substituting unit time and length. The displacement of the IWs located on the initial grid was accumulated over time to obtain cellular trajectories, which have accuracy close to the actual manual tracking method.

### 2.7. Path Length and Directional Persistence

In obtaining the cell trajectory, the displacements of each grid were accumulated over time. By this means, the total path length each cell traveled for 9 h was calculated. Moreover, directional persistence was identified by dividing the distance from the initial position to the end position of the cell by the path length so that the ratio was used to quantify whether the cell trajectory was close to a straight line.

### 2.8. Differentiation of Low or High Migratory Cells

In the distribution of cellular speeds measured within a cell island, the relative cell motility was spatially distinguished such that the highest 25% of cell speeds are indicated by black edge circles, the lowest 25% of cell speeds are indicated by gray edge circles, and the middle quartiles 25 to 75% are indicated by gray circles.

### 2.9. Immunoblotting

Total protein was extracted after adding Radioimmunoprecipitation assay (RIPA) lysis buffer (0.5% NP40, 150 mM NaCl, 0.5 mM EDTA, 20 mM Tris) with protease inhibitor cocktail, and 1 mM dithiothreitol (DTT). After quantification, the extracted protein was subjected to electrophoresis using acrylamide gel, and then transferred to the polyvinylidene fouoride (PVDF) membrane. Then, after blocking with 5% skim milk for 1 h, the primary antibody was reacted overnight at 4 °C, the secondary antibody was bound, and the result was measured using an X-ray film.

The following antibodies were used for western blot: ZO-1 (Invitrogen, Carlsbad, CA USA, 33-9100, 1:1000 dilution), claudin-1 (Invitrogen, 51-9000, 1:1000 dilution), E-cadherin (BD Biosciences, San Jose, CA, USA, 610154, 1:1000 dilution), β-catenin (BD Biosciences, 610182, 1:1000 dilution), claudin-6 (Abcam, Cambridge, UK, ab199670, 1:1000 dilution), claudin-2 (Santacruz Biotechnology, sc-293233, 1:1000 dilution), claudin-3 (Santacruz Biotechnology, sc-17662, 1:1000 dilution), claudin-4 (Santacruz Biotechnology, sc-17664, 1:1000 dilution), claudin-9 (Santacruz Biotechnology, sc-398836, 1:1000 dilution) and β-actin (Santacruz Biotechnology, sc-47778, 1:1000 dilution), claudin-7 (LSBio, Seattle, WA, USA, LS-B7594, 1:1000 dilution), Goat-anti-Rabbit igG HRP-conjugated antibody (BIO-RAD, Hercules, CA, USA, 170-6515, 1:10000 dilution), Rabbit-anti-goat igG HRP-conjugated antibody (BIO-RAD, 172-1034, 1:10000 dilution) and Goat-anti-mouse igG HRP-conjugated antibody (BIO-RAD, 172-1011, 1:10000 dilution).

### 2.10. In Vitro Wound Healing Assay

Cell migration was induced by scraping the cells at about 90% confluence with a 200 μL pipette tip and replacing them with fresh 10% FBS containing medium. Using Dino-eye Digital Eye Piece Camera, 0 h and 24 h after scratch were photographed, and the change in cell area was measured by Image J (NIH, Bethesda, MD, USA).

### 2.11. Quantitative RT-PCR

Total RNA was extracted using Trizol reagent (ambion by life technologies, Carlsbad, CA, USA) according to the manufacturer’s instructions. 1 µg of total RNA was used for cDNA synthesis with random hexamers. For RT-PCR amplification of ZO-1, claudin-1 and claudin-7 an initial amplification using ZO-1, claudin-1, and claudin-7 primers was done with a denaturation step at 94 °C for 5 min, followed by 40 cycles of denaturation at 94 °C for 30 s, primer annealing at 58 °C for 30 s, and primer extension at 72 °C for 30 s. Upon completion of the cycling steps, a final extension at 72 °C for 5 min was done and then the reaction was stored at 4 °C. Real-time PCR was carried out using an ABI 7500 Sequence Detection System (Applied Biosystems, Foster City, CA, USA). Reactions were run in triplicate in three independent experiments. The geometric mean of housekeeping gene β-actin was used as an internal control to normalize the variability in expression levels. The primer sequences are provided in the Supplementary (Table S1). Expression data were normalized to the geometric mean of housekeeping gene β-actin to control the variability in expression levels and were analyzed using the 2^−ΔΔCT^ method.

### 2.12. Immunofluorescence Staining

Cells were incubated on coverslips, fixed with 4% (PFA), permeabilized using 0.2% Triton X-100, and blocked in 1% BSA for 30 min at RT. The cells were then incubated with primary antibodies against ZO-1 (Invitrogen, 33-9100 and 61-7300, 1:200 dilution) and b-catenin (BD Biosciences, San Jose, CA, USA, 610154, 1:200 dilution) O/N at 4 °C. After washing three times with PBST (PBS containing 0.1% Tween20), Alexa Fluor 488 (Invitrogen, A11001 and A11008, 1:400 dilution), Texas red (vector, TI-2000 and TI-1000, 1:400 dilution), and phalloidin-TRITC (sigma, P-1951, 1:200 dilution) The bound anti-IgG secondary antibody for 1 hr was incubated at RT in the darkroom. After washing three times with PBST, the coverslip was attached to the sliding glass using ELVANOL^®^. Images were acquired on a ZEISS LSM 700 (Carl Zeiss, Jena, Thuringia, Germany) confocal microscope.

## 3. Results

### 3.1. E-Cadherin Re-Expression in AGS Gastric Cancer Cells Increases Cell Migration

Re-expression of E-cadherin has previously been shown to induce NF-κB-mediated cell proliferation to compensate for the suppression of Wnt signaling in AGS gastric cancer cells [27]. To evaluate the effect of re-expression of E-cadherin on cell migration, we analyzed cell motility with a cell island model in which the number and density of clustered cells were controlled using a pattern of homogeneous size and shape (Figure 1a). The cell island started at 1 mm in diameter and expanded by isotropic free-edge migration (Figure 1b). To identify the extent of expansion of clustered cells with or without E-cadherin, together with AGS cells, EC96 cells (AGS cells overexpressing E-cadherin) were used to compare how the movement of clustered cells changed over 9 h (Figure 1c–e). The cell islands expanded more widely in EC96 cells compared with AGS cells (Figure 1c). The cellular trajectory of individual cells over 9 h showed that cells hardly moved or meandered over the whole region of the AGS cell island (Figure 1d). On the other hand, cells in the EC96 cell island migrated from the center to the edge gradually and evenly. Since the EC96 cells at the edge of the island were more straightforward moving and migrated further than AGS cells, the increase in the area of EC96 cell islands was more than 1.5 times that of the AGS cell islands (Figure 1e). These results indicate that E-cadherin re-expression promoted collective cell migration.

### 3.2. E-Cadherin Re-Expression in AGS Gastric Cancer Cells Increases Fast and Straightforward Cell Migration

**Figure 2:**
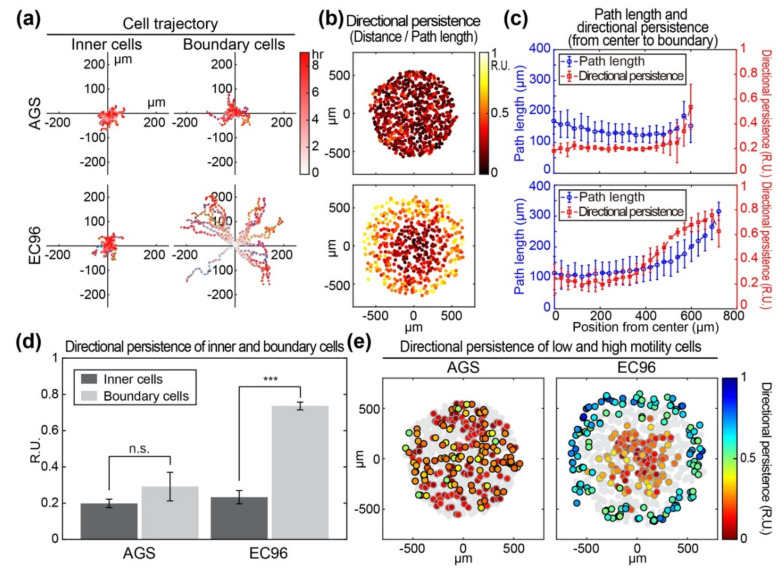
Comparison of motility and directionality of AGS and EC96 cell islands. (**a**) The trajectory from the initial locations of the inner and boundary cells within the AGS and EC96 cell islands. The stronger red color of the dots indicates more time passed. (**b**) Color-code map of the directional persistence of the cells within the AGS and EC96 cell islands. The brighter dots indicate higher directional persistence. (**c**) Average path length and directional persistence along with the same distance from center to edge of the AGS and EC96 cell islands. The error bars represent the standard deviations calculated from separate assays on each group (*n* = 5). (**d**) Average directional persistence of inner and boundary cells within the AGS and EC96 cell islands. The error bars represent the standard errors calculated from separate assays on each group (n.s. not significant and *** *p* < 0.001; *n* = 5). (**e**) Color-code map of the directional persistence of the lowest 25% (gray edge circles) and the highest 25% (black edge circles) of motile cells within the AGS and EC96 cell islands. Gray circles represent mid-quartile (25–75%) motile cells within each group population.

To analyze the mode of cell migration in detail, approximately 20 cells located in the center and the edge at the starting point were chosen and referred to as inner cells and boundary cells, respectively. Trajectories of inner and boundary cells were measured separately (Figure 2a). The inner cells appeared to meander in place within a range of 50 μm or less in both AGS and EC96 cell islands. However, boundary cells of the AGS island showed a similar meandering pattern of movement over a range of less than 100 μm. In the EC96 cell island, boundary cells showed increased linear movement over a distance of approximately 200 μm. Imaging of the production of reactive oxygen species (ROS) showed that most ROS-producing cells were located in the periphery of cell clusters (Figure S1), similar to leader cells of the collective migration model. To quantify the directional persistence of movement for each cell, the migration distance from the initial to the end position was divided by the total path length for 9 h and the ratio was expressed as a color (dark to bright) at each cell location at the endpoint (Figure 2b). In AGS cell islands, both inner and boundary cells showed a low directional persistence of 0.2 relative units (RU). In EC96 cell islands, inner cells had a low directional persistence with a value of approximately 0.2 RU, but the directional persistence increased at cell locations closer to the edge (~0.8 RU). In more detail, migration of inner and boundary cells was analyzed and compared based on cellular trajectory, directional persistence, and path length (Figure 2c). Most AGS cells showed a migration path length of approximately 150 μm regardless of location in the island, but the area of the island did not increase significantly due to a directional persistence value of 0.2 RU. In EC96 cells, migration path length and directional persistence increased from the center to the periphery of the cell island, from approximately 100 μm to 300 μm in path length and 0.2 RU to 0.8 RU in directional persistence. The increased path length and directional persistence of EC96 cells, especially in boundary cells, facilitated an expansion of the area of the EC96 island. In a quantitative comparison of directional persistence of inner and boundary cells, AGS cells showed an insignificant increase in directional persistence while boundary EC96 cells showed approximately 3.5-fold higher directional persistence than that of inner cells (Figure 2d). In the analysis of spatial distribution of motile cells, highly migratory cells in the upper 25% (black border) and minimally migratory cells in the lower 25% (gray border) of the population were displayed in the cell island and directional persistence was expressed in a color gradient (red to blue) (Figure 2e). In the AGS cell island, highly and minimally migratory cells were distributed evenly and directional persistence did not correspond to the degree of migration. In EC96 cells, highly migratory cells were predominantly located in the boundary of the cell island, suggesting that E-cadherin re-expressing cells acquired the ability to undergo fast and straightforward migration.

### 3.3. E-Cadherin Expression Regulates TJ Protein Expression and Cell Migration

**Figure 3:**
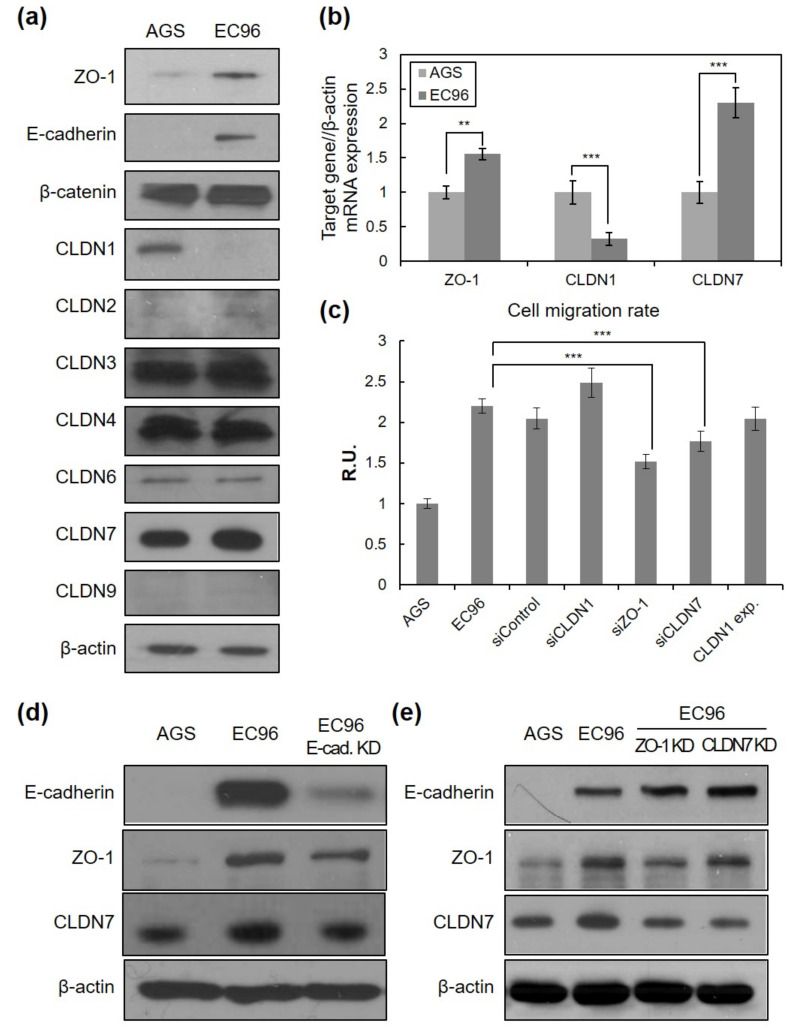
Tight junction proteins are related to cell migration. (**a**) Cell lysates were subjected to immunoblot analysis for ZO-1, E-cadherin, β-catenin, CLND-1, -2, -3, -4, -6, -7, -9, and β-actin. (**b**) Total RNA extracted from AGS and EC96 gastric cancer cells were subjected to RT-PCR with ZO-1, CLDN1, and CLDN7-specific primers. Averages of three independent experiments with error bars are presented. ** *p* < 0.01; *** *p* < 0.001. (**c**) EC96 cells were transfected with 100 nM of ZO-1, CLDN1, CLDN7 siRNA (“siZO-1, siCLDN1, siCLDN7) or scramble control siRNA (“siControl”) or 1 μg of the CLDN1 expression vector. In vitro cell migration was tested by the wound healing assay. Averages of five independent experiments with error bars are presented. *** *p* < 0.001. (**d**) AGS, EC96, and E-cad. KD (E-cadherin-knockdown EC96) cell lysates were subjected to immunoblot analysis for E-cadherin, ZO-1, CLDN7, and β-actin. (**e**) AGS, EC96, ZO-1 KD (ZO-1-knockdown EC96), and CLDN7 KD (CLDN7-knockdown EC96) cell lysates were subjected to immunoblot analysis for E-cadherin, ZO-1, CLDN7, and β-actin.

To elucidate the underlying mechanism of enhanced migration of EC96 cells, adhesion molecules were analyzed. Expression levels of some TJ-related proteins were noticeably increased. Immunoblotting revealed increased ZO-1 and CLDN7 levels, while CLDN1 expression was decreased in EC96 cells compared with AGS cells (Figure 3a). These results were confirmed with quantitative RT-PCR (Figure 3b), suggesting that the expression of E-cadherin regulates the transcription of some TJ genes. In a transient knockdown experiment using ZO-1 and CLDN siRNAs (siZO-1 and siCLDN7, respectively), both siRNA transfections significantly reduced cell migration, which was measured with a scratch assay at 48 h after the introduction of siRNA (Figure 3c). In the case of CLDN1, neither transient expression of siRNA nor cDNA targeting CLDN1 affected cell migration.

To confirm whether E-cadherin expression affects TJ protein expression and cell migration, a cell line with knockdown of E-cadherin (EC96/E-cad KD) was established from EC96 cells. Reduction of ZO-1 and CLDN7 expression could be observed as E-cadherin expression was reduced (Figure 3d). Results of quantitative RT-PCR supported these results (Figure S2a). EC96/E-cad KD cells also showed reduced straightforward movement (Figure S3). EC96/E-cad KD cell islands were less expanded than EC96 cell islands when the movement of clustered cells was observed for 9 h (Figure S3a,b). In trajectory analysis, movement in a zigzag pattern more similar to that of AGS cells than EC96 cells was observed. The directional persistence of boundary cells of the EC96/E-cad KD cell line was also reduced compared with that of EC96 cells (Figure S3c,d). Analysis of migration and directional persistence revealed that highly and minimally migratory cells of the EC96/E-cad KD cell line were distributed in a jumbled manner, more similar to AGS cells than EC96 cells, and cells with different degrees of directional persistence were also mixed in the distribution (Figure S3e).

Further experiments using ZO-1 knockdown EC96 cells showed a reduction of CLDN7 expression while the effect of CLDN7 knockdown on ZO-1 was unclear (Figure 3e, Figure S2b). E-cadherin expression was unaffected in both knockdown cell lines. These results suggest that expression levels of various TJ proteins are cross-regulated following a significant change in a cellular context such as E-cadherin expression and adhesive properties, which consequently regulates cellular phenotypes such as straightforward movement.

### 3.4. ZO-1 Is Involved in Regulation of Cell Migration

**Figure 4:**
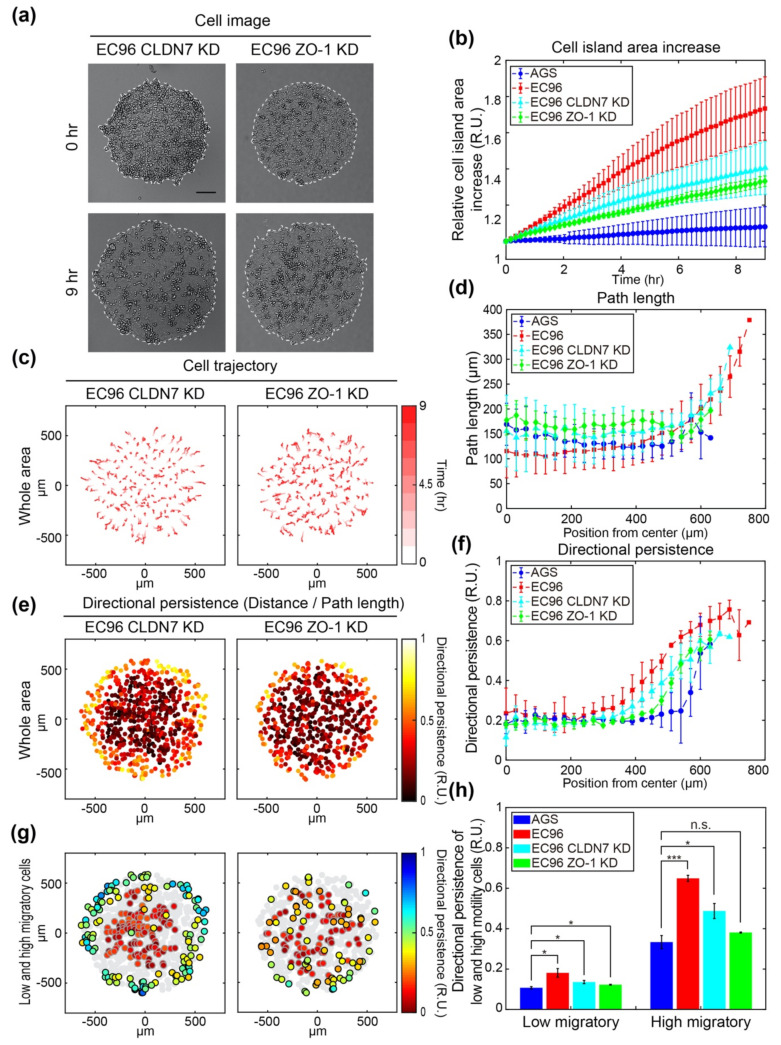
Analysis of motility and directionality of cell islands composed of tight junction protein knockdown cell lines and comparison with AGS and EC96. (**a**) Bright-field images of CLDN7 and ZO-1 KD cell islands at 0 h and 9 h after release (scale bar = 200 μm). (**b**) Relative area increase of cell islands composed of the AGS (blue circle), EC96 (red square), CLDN7 KD (cyan triangle), and ZO-1 KD (green diamond) cells over time. The error bars represent the standard deviations calculated from separate assays on each group (*n* = 5). (**c**) The trajectory of the cells within the CLDN7 KD and ZO-1 KD cell islands. The stronger red color of the arrows indicates more time passed. (**d**) Average path length along the same distance from center to edge of the AGS (blue circle), EC96 (red square), CLDN7 KD (cyan triangle), and ZO-1 KD (green diamond) cell islands. The error bars represent the standard deviations calculated from separate assays on each group (*n* = 5). (**e**) Color-code map of the directional persistence of the cells within the CLDN7 KD and ZO-1 KD cell islands. The brighter dots indicate higher directional persistence. (**f**) Average directional persistence along the same distance from center to edge of the AGS (blue circle), EC96 (red square), CLDN7 KD (cyan triangle), and ZO-1 KD (green diamond) cell islands. The error bars represent the standard deviations calculated from separate assays on each group (*n* = 5). (**g**) Color-code map of the directional persistence of the low 25% (gray edge circles) and high 25% (black edge circles) of motile cells within the CLDN7 KD and ZO-1 KD cell islands. Gray circles represent mid-quartile (25–75%) motile cells within each group population. (**h**) Average directional persistence of low and high motility cells within the AGS (blue), EC96 (red), CLDN7 KD (cyan), and ZO-1 KD (green) cell islands. The error bars represent the standard errors calculated from separate assays on each group (n.s. not significant, * *p* < 0.05 and *** *p* < 0.001; *n* = 5).

To determine the effects of TJ proteins such as ZO-1 and CLDN7 on cell movement, ZO-1, and CLDN7 knockdown (ZO-1 KD and CLDN7 KD) cells were subjected to a migration assay using the cell island expansion model (Figure 4). The increases of both ZO-1 KD and CLDN7 KD cell islands after 9 h were intermediate between those of AGS and EC96 cells, and the increase in the CLDN7 KD cell island was slightly higher than that of the ZO-1 KD cell island (Figure 4a,b), suggesting that ZO-1 and CLDN7 regulate cell migration following E-cadherin re-expression. In trajectory analysis, CLDN7 KD cells migrated from the center to the edge, similar to EC96 cells, whereas ZO-1 KD cells exhibited a zigzagging motion, similar to AGS cells (Figure 4c). Analysis of the path lengths of cells according to distances from the center of the island revealed that both CLDN7 KD and ZO-1 KD cells inside of islands (0–450 μm) showed a migration path length similar to that of AGS cells. However, the periphery of the CLDN7 KD cell island (>450 μm) showed that the migration length gradually increased as the cells got further from the center, similar to EC96 cell islands (Figure 4d). In addition, the difference between the central and peripheral directional persistence of cells in the CLDN7 KD cell island was more distinctive than in the ZO-1 KD cell island (Figure 4e). The directional persistence of CLDN7 KD and ZO-1 KD cells was similar from the center of the cell island to approximately 300 μm, but near the edge, CLDN7 KD cells showed slightly higher directional persistence values compared to ZO-1 KD cells (Figure 4f). Furthermore, the spatial distribution of motility and directional persistence showed a more distinctive distribution in CLDN7 KD cell islands than in ZO-1 KD cell islands (Figure 4g). Cells located at the periphery of the CLDN7 KD cell island showed higher migration speed and directional persistence than cells located at the center, while ZO-1 KD cells did not show the spatial distribution of migratory cells. Comparison of directional persistence in low and high migratory cells showed that the cells with low speed showed low directional persistence in all conditions at values between 0.1 and 0.2 RU, and the differences between AGS cells and other cell types had low significance (Figure 4h). However, the cells with high speed showed higher directional persistence values between 0.3 and 0.7 RU. In particular, EC96 cells exhibited a significant difference compared with AGS cells, indicating a distinct correlation between speed and directional persistence. Knockdown of ZO-1 and CLDN7 affected the directional persistence of cells, indicating that the correlation between speed and directional persistence of ZO-1 KD cells was lowered, similar to that of AGS cells.

### 3.5. ZO-1 Regulates Directional Movement of Cells

**Figure 5:**
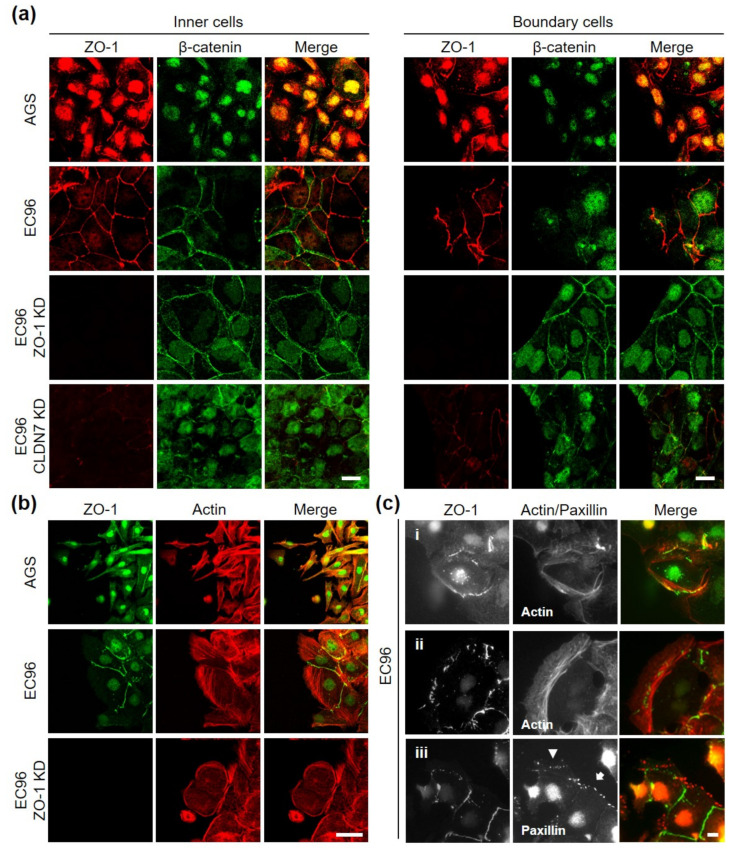
Analysis of differential expression of ZO-1 protein in migratory cells. (**a**) Double-immunofluorescence labeling for β-catenin (green) and ZO-1 (red) in inner cells (left panel) and boundary cells (right panel) of AGS, EC96, and ZO-1-knockdown EC96 cells. Scale bar: 100 µm. (**b**) AGS, EC96, and ZO-1-knockdown EC96 cells were subjected to immunofluorescence staining for ZO-1 (green) and Actin (red) at the leading edge. Scale bar: 100 μm. (**c**) EC96 cells were subjected to double-immunofluorescence labeling for ZO-1/Actin (c-i) and (c-ii) or ZO-1/Paxillin (c-iii) at the leading edge. Scale bar: 100 μm.

To investigate the underlying mechanism, an immunofluorescence assay was conducted (Figure 5). We analyzed β-catenin instead of E-cadherin because β-catenin also reflected E-cadherin-mediated AJ structure but showed more informative and clear images than E-cadherin labeling in our system. In AGS cells, most β-catenin was expressed in the nucleus and was difficult to observe in the cell membrane, while a small fragmented linear pattern of ZO-1 labeling was observed in cells regardless of whether they were inner or boundary cells (Figure 5a). In EC96 cells, β-catenin and ZO-1 were clearly located in the cell membrane, appearing like a net in inner cells. However, in boundary EC96 cells, membrane β-catenin appeared to be decreased and some nuclear expression was observed, while ZO-1 was still expressed in the membrane. In the case of ZO-1 KD cells, β-catenin labeling in boundary cells appeared like a net, similar to inner cells. Expression of E-cadherin was not affected by ZO-1 knockdown (Figure 3e), and β-catenin functions appeared to be enhanced in the absence of ZO-1.

To analyze the detailed relationship between ZO-1 expression and cell movement direction, double labeling for actin and ZO-1 was performed (Figure 5b). Linear patterns of ZO-1 were observed on opposite sides of lamellipodia protrusions at cell contact sites with posterior cells in EC96 cells. In ZO-1 KD cells, the frequency of lamellipodia protrusions was reduced as cell migration decreased (see Figure 3c). In time-lapse analysis, EC96 cells appeared to move forward while maintaining cell-cell contact with posterior cells, while ZO-1 KD cells moved in place with reduced forward migration (Video S1). In detailed observations of front-line cells, some ZO-1 dots could be observed at the site of assembling actin filaments (Figure 5c(i)), and as actin filaments elongated, these ZO-1 spots remained at the base of lamellipodia protrusions (Figure 5c(ii)). Similarly, ZO-1 dots formed around focal adhesion plaques (Figure 5c(iii), arrow), and as lamellipodia protruded further, ZO-1 dots were observed behind newly formed focal adhesion plaques (arrowhead). These results suggested that ZO-1 can be located not only at the posterior cell-cell contact sites of active migrating cells but also at the base of newly protruding lamellipodia. These observations of ZO-1 localization led us to speculate that cellular tensions according to cell movement produced compensating reactional force at cell-cell and/or matrix adhesion sites and ZO-1 may be located at the site where cellular tension-traction is generated, which could regulate cell movement direction.

## 4. Discussion

Collective cell migration of epithelial cancer is an important mechanism of metastasis. Cell-cell and cell-matrix interaction are one of the key factors governing migration. To demonstrate enhanced cell migration of cancer cells, especially in terms of directional movement, we used circular-shaped cell island patterning for free edge migration and measured cell movements according to space-time by analyzing cellular kinematics. The model enabled us to analyze the migratory activity of cells using detailed variables such as directional persistence of individual cells, distribution of highly and minimally migratory cells in the cell island, and correlation between migratory variables. Cell island with epithelial cancer cells expressing E-cadherin expanded >1.5 times more rapidly than the normal AGS cell island. Trajectory analysis to trace the movements of individual cells showed that E-cadherin expressing cells moved more straightforwardly to the free space. Although the major function of E-cadherin is known as a modulator of cell migration, there are debates regarding the functional role of E-cadherin in cancer metastasis and migration [28,29]. Downregulation of E-cadherin expression ensures that static epithelial cells convert to dynamic mesenchymal cells [30,31]. However, the expression of E-cadherin in metastatic cancer cells is essential for re-establishing a tumor mass in a new organ environment. Cells also exhibit mesenchymal characteristics by promoting the transcription of E-cadherin during the mesenchymal epithelial transition (MET) [32,33]. Downregulation of E-cadherin in highly motile cells blocks their migration [15,34]. These results suggest that E-cadherin actively participates in the regulation of cell migration. Our results showed that knockdown of E-cadherin expression reduced migration and directional persistence of EC96 cells. These results suggest that the expression of E-cadherin facilitates cell migration by regulating directional cell migration together with increased speed of movement.

ZO-1 has been proposed to act as a scaffolding protein in the assembly of TJs and can form a link between AJs and TJs [35]. In epithelial cells, ZO-1 is concentrated at TJs through direct binding of claudins [36], occludins [37,38], and junctional adhesion molecules (JAMs) [39,40]. ZO-1 has also been shown to colocalize with cadherins through direct or indirect interaction with α-catenin [41]. These findings suggest that ZO-1 plays a key role in the establishment of TJs and AJs. In our study, AGS cells, which have low E-cadherin expression, showed some junctional ZO-1 protein expression, which showed a tendency to increase as cells approached confluence. Considering the low adhesiveness of AGS cells, these ZO-1-containing structures appeared to not exhibit classical TJ functions, including barrier function. Following the expression of E-cadherin in AGS cells, the expression and junctional localization of ZO-1 was clearly increased, indicating that E-cadherin can promote the assembly of TJs containing ZO-1. Similar results were previously reported in which restored E-cadherin in AGS cells formed fully developed epithelial sheets with proper TJs and AJs containing β-catenin, p120-catenin, nectin-1, afadin, and ZO-1 [42]. In this study, we investigated whether E-cadherin-enhanced directional movement of cells could be mediated by ZO-1 and TJ-related protein expression. Many lines of evidence have shown that downregulation of TJ proteins such as claudins, occludin, and ZO-1 reduced cell migration [43,44,45]. Enhanced motility of EC96 cells was accompanied by increased expression of ZO-1 and CLDN7 at the protein and transcript levels, while CLDN1 expression was reduced in EC96 cells (Figure 3). Given that overexpression of CLDN1 had little effect on EC96 cell migration (data not shown), we focused on elucidating the roles of ZO-1 and CLDN7 in EC96 cell motility. Both ZO-1 and CLDN7 knockdown resulted in a significant reduction of EC96 cell migration and directional persistence of cell movement (Figure 4). These results suggest that E-cadherin-induced enhancement of cell migration is also mediated by the TJ proteins ZO-1 and CLDN7 in AGS cells.

Leader cells at the front of the group drive migration while follower cells communicate with leader cells or other follower cells to coordinate migratory cell responses during collective migration [46,47]. EC96 cells expressing E-cadherin showed the increased migratory activity as well as directionality. Cells in the front line of the EC96 cell group also showed increased ROS production, suggesting that oxidative respiration is dominant in the leader cells, which is correlated with our previous results (Figure S1) [27]. The metabolic switching is also observed in other studies [48]. Our results suggest that the boundary cells analyzed in our model showed leader cell characteristics by increasing energy generation and migratory activity. The expression of ZO-1 in the boundary cells was shown at cell contact sites while β-catenin was absent at these sites (Figure 5a), suggesting that ZO-1-containing structures are more engaged in intercellular communication with follower cells than β-catenin-containing structures (AJs). Given our evidence that ZO-1 is involved in the regulation of migratory speed and straightforward movement (Figure 4), we speculated that ZO-1 in boundary cells is involved in cell migration as well as directionality of cell group. Many recent studies have suggested that follower cells are required for efficient migration; these follower cells control the leader cells by regulating polarization of the entire cell group and supracellular contraction [47,48]. Our result showed that the band of ZO-1protein is localized and aligned perpendicular to the direction of actin-based protrusion in EC96 cells. As ZO-1 can regulate actin organization directly and/or indirectly [49], we can speculate that expression of ZO-1 plays a role in regulating migratory speed and directionality through regulation of actin organization where cellular tension is generated against traction force as the cell membrane protrudes. Collective cell migration of cancer epithelial cells is governed by cell-cell junction in leader-follower cells. ZO-1 as a mediator of TJ plays a key role in the interaction of leader-follower cells by localizing and aligning at the contact point. Our results suggest that migratory activity and directionality during collective cell migration could be controlled by the interaction of leader-follower cells by ZO-1 protein.

## 5. Conclusions

Analysis of EC96 cells derived from AGS gastric cancer cells showed that E-cadherin re-expression enhanced cell migration speed and straight movement through regulation of tight junction (TJ) protein, ZO-1, and claudin-7 expression. In the periphery of cell clusters, TJ structures appeared to play important roles in intracellular communication. Localization of ZO-1 at the base of protruding lamellipodia and cell contact sites at the rear of cells implied that ZO-1 might be linked to the interaction between traction and tensile forces. In conclusion, E-cadherin plays a positive role in cell migration through the regulation of TJ proteins in cells with malignant phenotypes.

## Data Availability

Data is contained within the article or Supplementary Materials.

## Supplementary Materials

The following are available online at https://www.mdpi.com/article/10.3390/bioengineering8050065/s1, Figure S1: Detection of ROS in migrating cells. Figure S2. RT-PCR of EC96 and various knock-down cells. Figure S3: Expansion, motility, and directionality of EC96 E-cadherin knock-down cell islands, Table S1: Forward and reverse primers for real-time quantitative RT-PCR, Video S1: Time-lapse microscopy of cell migration.
