# Supplementary material for: Expression of E-Cadherin in Epithelial Cancer Cells Increases Cell Motility and Directionality through the Localization of ZO-1 during Collective Cell Migration
Source: Bioengineering (Basel). 2021 May 11;8(5):65. doi: 10.3390/bioengineering8050065 (PMC8151941; doi:10.3390/bioengineering8050065)
Supplement: Supplementary file 1 [file bioengineering-08-00065-s001.zip › Supplementary Materials_210505.pdf]

Supplementary Materials

Expression of E-cadherin in epithelial cancer cells increases cell motility and directionality through the localization of ZO-1 during collective cell migration

Song Yi Park[1†], Hwanseok Jang[2†], Seon Young Kim[1], Dasarang Kim[1], Yongdoo Park[2*] and Sun-Ho Kee[1*]

[1]Department of Microbiology, College of Medicine, Korea University, Seoul, Korea
[2]Department of Biomedical Sciences, College of Medicine, Korea University, Seoul, Korea

[†] Authors contributed equally to this work.

[*]Corresponding authors: Yongdoo Park and Sun-Ho Kee
E-mail: ydpark67@korea.ac.kr; keesh@korea.ac.kr

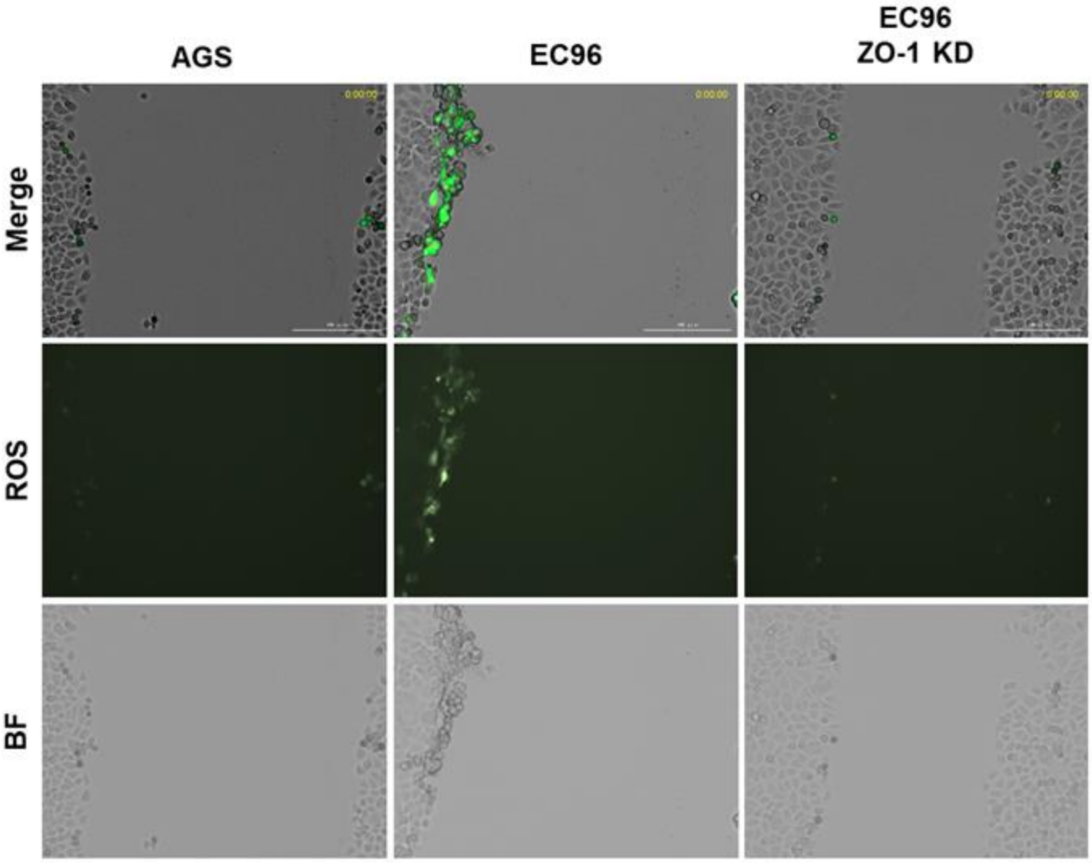

Figure S1. Detection of ROS in migrating cells. Intracellular ROS levels were also observed by live cell imager (magnification, ×100). The green fluorescence of DCF represents the levels of intracellular ROS.

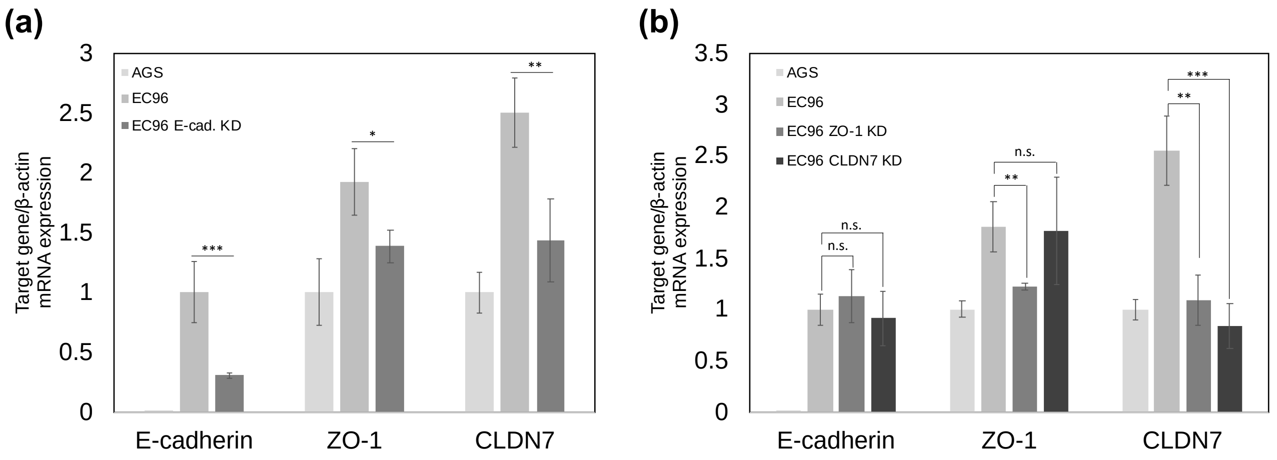

Figure S2. Re-expression of E-cadherin regulates ZO-1 and CLDN7 expression. (a) Total RNA extracted from AGS, EC96 and EC96 E-cad. KD cells were subjected to RT-PCR with E-cadherin, ZO-1 and CLDN7-specific primers. (b) Total RNA extracted from AGS, EC96, EC96 ZO-1 KD and EC96 CLDN7 KD cells were subjected to RT-PCR with E-cadherin, ZO-1 and CLDN7-specific primers. Averages of three independent experiments with error bars are presented. *P<0.05; **P<0.01; ***P<0.001; n.s., not significant.

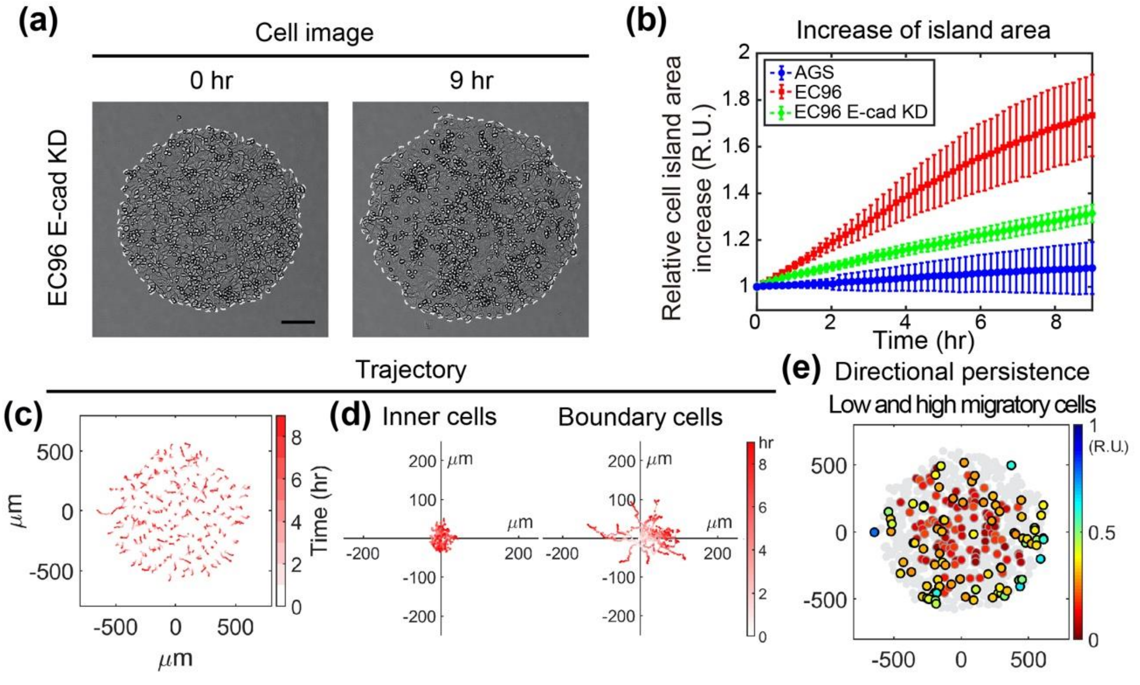

Figure S3. Analysis of motility and directionality of cell islands composed of E-cadherin knockdown cell line and comparison with AGS and EC96. (a) Images of E-cadherin KD cell islands at 0 hr and 9 hr after release (scale bar = 200 μm). (b) Relative area increase of cell islands composed of the AGS, EC96 and E-cadherin KD cells over time (n = 5). (c) Trajectory of the cells within the E-cadherin KD cell island. (d) Trajectory from the initial locations of the inner and boundary cells within the E-cadherin KD cell island. (e) Color-code map of the directional persistence of the lowest 25% and the highest 25% of motile cells within the E-cadherin KD cell island. Gray circles represent mid-quartile (25 – 75%) motile cells within each group population.

Table S1. Forward and reverse primers for real-time quantitative RT-PCR

Gene	Forward primer	Reverse primer
E-cadherin	ACC ATT AAC AGG AAC ACA GG	CAG TCA CTT TCA GTG TGG TG
ZO-1	TGC CAT TAC ACG GTC CTC TG	GGT TCT GCC TCA TTT CCT C
CLDN1	TTC TCG CCT TCC TGG GAT G	CTT GAA CGA TTC TAT TGC CAT ACC
CLDN7	TGA GAG CAA GGC TGG GTA C	TGG GAA TGA ATG TCG AGA TAC G
β-actin	ATC TAC GAG GGG TAT GCC	TAG CTC TTC TCC AGG GAG

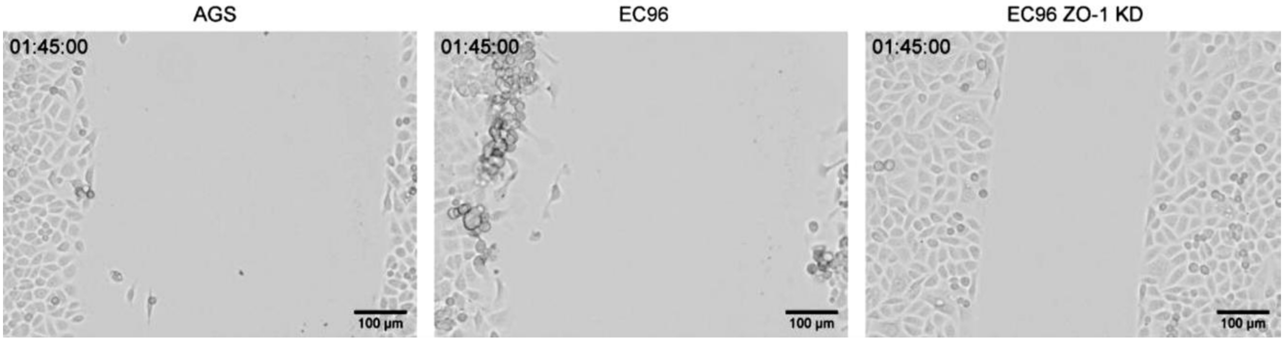

Vides S1. Time-lapse microscopy of cell migration
